# Near-Infrared Autofluorescence to Improve Visualization During Adrenal Surgery: A New Frontier for Interdisciplinary Collaboration

**DOI:** 10.1007/s00268-022-06820-6

**Published:** 2022-11-09

**Authors:** Julie A. Miller

**Affiliations:** Department of Surgery, The Royal Melbourne Hospital Endocrine Surgery Unit, The University of Melbourne, Melbourne, Australia

Adrenalectomy is a relatively low-volume endocrine surgical procedure, with an estimated 6000 adrenalectomies per year in the USA compared with 130,000 thyroidectomies [1, 2] In the USA, 80% of adrenalectomies are performed by low-volume adrenal surgeons(< 6 per annum), and the median annual surgeon volume is one case [3]. Differentiating adrenal tissue from perinephric fat can be challenging even for high-volume surgeons, particularly in overweight or obese patients.

Near-infrared autofluorescence (NIRAF) is a label-free modality being investigated as a method to better differentiate adrenal tissue from perinephric fat, potentially improving surgical precision during minimally invasive adrenalectomy. Improved visualization could be particularly helpful for low-volume surgeons, or in patients with excess visceral fat. This paper by Thomas et al. [4], a collaboration between surgeons and engineers, is an elegant pilot study investigating the optical characteristics of normal and pathologic adrenal glands compared with surrounding structures, using commercial and custom-built cameras and handheld probes to assess fluorescence.

The authors used ten frozen tissue bank specimens and fifty prospectively collected adrenal specimens to quantitatively analyse NIRAF characteristics of normal and pathologic adrenal specimens ex vivo and trialled a first-of-its-kind custom-built fibreoptic probe and spectroscopy system to assess four adrenal glands in vivo during open nephrectomy.

The excitation wavelength of 785 nm used in handheld probe-based system (PTeye) and the custom-build camera device resulted in better NIRAF to background ratio (NBR) than the commercially available camera using 760 nm excitation wavelength. The emission peak was at 820 nm. The authors found the highest autofluorescence and NBR in normal adrenal cortex and adrenocortical adenomas, regardless of secretory status. Fluorescence was patchy and heterogenous in pheochromocytomas and negligible in primary adrenal malignancy, cysts, and myelolipomas. Normal adrenal cortex had significantly higher fluorescence than medullary tissue or malignant tumours, allowing demarcation of malignant or medullary tumours from normal adrenal cortex. Lastly, normal adrenal cortex and adrenocortical adenomas fluoresced at a much higher level than surrounding perinephric fat, vessels, and the kidney.

In summary, Thomas et al. demonstrated strong label-free autofluorescence in normal adrenal cortex and benign cortical adenomas, moderate patchy fluorescence in phaeochromocytomas, and minimal to no fluorescence in surrounding fat or primary adrenal malignancy. The best excitation wavelength was 785 nm, and emission peak was 820 nm. A handheld probe-based system (PTeye) yielded better differentiation than a camera-based NIR system (PDE-Neo II). These findings were independent of specimen temperature or blood supply.

NIRAF may prove to be a useful adjunct in adrenal surgery, but this field of study is in its infancy, and the practical utility of NIRAF for adrenal surgery is yet to be demonstrated. Advantages over alternative adjuncts for adrenal visualization include no requirement of infusion of a labelling substance such as indocyanine green or interrupting the procedure to introduce an ultrasound probe. Disadvantages include the lack of a commercially available system to detect autofluorescence during minimally invasive surgery, as well as potential cost of such a device once it is developed. It remains to be seen if such cost would be offset by improved outcomes. It also appears the ability to detect fluorescence decreases with increasing thickness of overlying fat and is no longer apparent through 8 mm of fat. If this limitation cannot be overcome, it would limit the utility of NIRAF for patients in which the technology would be most useful.

It appears that adrenal NIRAF is an adjunct that will be most useful to identify normal, intensely fluorescing adrenal cortex the surgeon wishes to preserve, for example when performing partial adrenalectomy for pheochromocytoma, rather than adrenal tissue the surgeon wishes to resect, in which case the entire fatty envelope is resected in order to avoid breaching the soft and friable adrenal capsule, particularly in cases of suspected malignancy when the noise to background ratio is smaller and the consequences of a capsular rupture infinitely higher. It is feasible but not definitively demonstrated that NIRAF could aid in identifying the optimal tissue plane for dissection.

This study is an important pilot exploring a new technology and examining how NIRAF in might be useful for the adrenal surgeon to improve the care we offer our patients. The methodology is logical and intuitive. The authors are creative and innovative and should be congratulated on their pioneering work. They demonstrate the benefits of cross-discipline collaboration between clinicians and biomedical engineers and may be opening a whole new frontier for scientific investigation in the field of ‘biophotonics’. The nuances and utility remain to be elucidated, but the kernel has been planted.

## References

[1] Murphy MM, Witkowski ER, Ng SC (2010). Trends in adrenalectomy: a recent national review. Surg Endosc.

[2] Sosa JA, Hanna JW, Robinson KA, Lanman RB (2013). Increases in thyroid nodule fine-needle aspirations, operations, and diagnoses of thyroid cancer in the United States. Surgery.

[3] Thomas SM, Adam MA, Pontius LN, Stang MT, Scheri RP, Roman SA, Sosa JA (2018). Each procedure matters threshold for surgeon volume to minimize complications and decrease cost associated with adrenalectomy. Surgery.

[4] Thomas G, Kiernan CM, Willmon PA (2022). Label-free enhancement of adrenal gland visualization using near-infrared autofluorescence for surgical guidance. World J Surg.

